# The predictive value of high school grade point average to academic achievement and career satisfaction of dental graduates

**DOI:** 10.1186/s12903-021-01662-5

**Published:** 2021-06-12

**Authors:** Ayah A. Al-Asmar, Yara Oweis, Noor H. Ismail, Alaa H. A. Sabrah, Islam M. Abd-Raheam

**Affiliations:** Department of Conservative Dentistry, School of Dentistry, University of Jordan, Amman, Jordan

**Keywords:** Grade point average, Academic, Dental, Student, Career

## Abstract

**Background:**

In a country where admission to dental schools is based solely on the cognitive abilities of students, the aim of the study was to investigate the correlation between high school grade point average and graduating academic achievement for dental students; moreover, determine whether a correlation exists between dental students’ graduating academic achievement and their career choices and job satisfaction.

**Methods:**

A five-year retrospective cohort study was conducted at the University of Jordan, involving (828) dental graduates first enrolled between 2010 and 2014. Correlations comparing high school grade point average and graduating academic achievement were done for the total sample composed of (736) students. A short survey was constructed to assess the career choices and job satisfaction for recently graduated dentists and correlate them with their graduating academic achievement.

**Results:**

Statistically significant but weak positive correlation (0.3) was found between high school grade point average and graduating academic achievement for dental students (*p* ≤ 0.05). Statistically significant correlations were found between graduating academic achievement and career choices and job satisfaction.

**Conclusion:**

The significant positive correlation between the high school grade point average and graduating academic achievement of our dental students indicate that our school admission system depending on high school grade point average is a valid system. There was a significant negative correlation between the graduating academic achievement and both the career choices and job satisfaction among fresh graduate dentists in Jordan. This information is required to update the dental school admissions procedures in response to the changing dental educational landscape.

**Supplementary Information:**

The online version contains supplementary material available at 10.1186/s12903-021-01662-5.

## Background

Intellectual abilities and cognitive components are fundamental elements for a successful dentist. The role of these cognitive factors in the prediction of academic performance and professional success is controversial [1].

Dental schools are faced with an ongoing challenge of selecting the most qualified students to fill a limited number of openings each year, which adds pressure on the educators to select prospective students based on cognitive and noncognitive merits thus ensure fewer students leave the program prematurely and a higher standard of dentists are produced [2]. It is thought that the predictive admission procedures can reduce dropout rates, improve the average academic performance, and selectively exclude applicants who are unlikely to be successful as practitioners [3]. Such situation generates high competition among students for admission [4].

Historically it has been commonplace for medical schools to use a variety of methods which examine a range of factors associated with success in medical school to select their students [5]. Systemic reviews’ findings concerning selection tools in medical education supported the fact that previous academic performance is a predictor of success [6]. There is evidence demonstrating that overall college grade point averages (GPA), as well as the science GPA, are the best predictors of success in dental schools, in spite that those GPAs do not necessarily indicate dental students’ success in terms of clinical performance on regional examinations [7].

The selection of students for admission in most dental schools in many countries rely on student’s high school cumulative grade point average (HS-GPA) as their sole criterion. Others add a specialized test like the Dental Admission Test (DAT), Graduate Australian Medical School Admissions Test (GAMSAT), and Saudi National Aptitude Exam (SAT1) and Saudi National Achievement Exam (SAT2) [4, 6, 8, 9]. However, the literature has highlighted that the predictive validity of admission tests for universities is still a matter of debate and controversy [4].

In Jordan HS-GPA is based on the final exam scores of the final twelfth high school year. The high school has two divisions, Scientific and Theoretic streams. The Ministry of Higher Education in Jordan and the Jordan Dental Association mandate that individuals enrolling in dental schools should graduate from the scientific stream, with a minimum HS-GPA of 85%.

The School of Dentistry at the University of Jordan uses the high school Grade Point Average HS-GPA as a tool for selecting the dental students, a tool readily identified in most educational systems. Yet evidence for the predictive validity of this method is sparse at best!

GPA is not just a frequently used measure of achievement in the selection of applicants for educational programs and graduate schools but also among job recruiters [10]. The aim is to deliver professionals who would add value to the job market and become future leaders in their field [11]. Employers have argued that grades are useful predictors because they reflect intelligence, motivation, and other abilities applicable to the job [12]. On the other hand, many academics have contended that grades are not good predictors of job performance [13, 14].

Career/Job satisfaction has been described as a “positive emotional state resulting from the appraisal of one’s job or job experience [15].” Although the literature has extensive research on career/job satisfaction and job performance of dentists [16–18], there is scarce research into job satisfaction among health service workers in the middle east in general and in Jordan in particular, and specifically among dentists. Besides, to the best of our knowledge, there are no studies assessing the relationship between academic variables and career choices and job satisfaction (CC-JS) of recently graduated dentists in Jordan and in the region.

The main objective of the current study was to examine the predictive validity of the admission criterion for dental students’ HS-GPA of their academic performance. Our specific objectives were to investigate the correlation between the HS-GPA and the graduating cumulative academic achievement (AA-GPA) of these students. In addition, the study aimed at assessing the distribution and the level of CC-JS among freshly graduated dentists in Jordan and the correlation between dental students’ AA-GPA and their CC-JS.

Our first null hypothesis: there is no correlation between dental students' HS-GPA and their graduating cumulative AA-GPA.

Our second null hypothesis: there is no correlation between dental students' AA-GPA and their CC-JS.

## Methods

According to the ethics policy of the University of Jordan, ethical approval form and permission to collect the needed data was signed and approved from the Faculty of Dentistry Research and Ethics Committee (FDREC) and the Institutional Review Board (IRB) at the University of Jordan.

Available HS-GPA were collected for dental students who were enrolled in the School of Dentistry at the University of Jordan, Amman, Hashemite Kingdom of Jordan, from year 2010 to year 2014. The sample size was of (828) students. Ninety-two students (n=92) who held non-Jordanian high school certificate was excluded, to ensure that all students hold Jordanian HS-GPA. The entry pathways for the excluded students use selection criteria not directly comparable with the standard entry procedures. The sample size after exclusion was (736) students, (598) females (81.25%) and (138) males (18.75%). Students with Jordanian high school certificate should score a mandatory minimum HS-GPA of 95.5% on average to compete for a place in the School of Dentistry. Nevertheless, students can still get accepted in dental school with HS-GPA less than 95.5% entering through either the international program or governmental scholarships.

The graduating academic achievement AA-GPA of dental students uses a 4.0-point GPA scale, which theoretically ranges according to the grading system from 1 to 4 as demonstrated in Table 1. Students are divided into two groups (HS-GPA ≥ 95.5% and HS-GPA < 95.5%) and their AA-GPA distribution is shown in Table 2.

**Table 1:** The graduating AA-GPA scale in School of Dentistry at the University of Jordan

AA-GPA	Degree
≥ 3.65	Excellent
3–3.64	Very good
2.5–2.99	Good
2–2.49	Acceptable
< 2	Weak

**Table 2:** The distribution of students according to their HS-GPA and AA-GPA on the grading system of the School of Dentistry at the University of Jordan

HS-GPA	AA-GPA excellent	AA-GPA Very good	AA-GPA good	AA-GPA acceptable	AA-GPA weak	Students (n = 736)
≥ 95.5%	32	194	204	33	0	463 (63%)
< 95.5%	3	61	141	68	0	273 (37%)

To assess career satisfaction and professional characteristics for our recently graduated dentists, a short electronic survey comprising of twelve questions was generated using Google forms and distributed electronically online to 525 fresh graduate dentists who graduated from the School of Dentistry at the University of Jordan between 2015 and 2019 and whom we could reach via e-mails and multiple social media platforms.

The survey consisted of 12 questions covering demographic and professional background characteristics and attitude towards job such as current occupation, level of satisfaction, work engagement’s rating, and career insights. The survey questions are demonstrated in Additional file 1.

With active informed consent of all participants, survey outcomes were matched with HS-GPA and AA-GPA as archived in the university’s files. A small pilot study was done to measure the reliability and validity of the questionnaire. All data collected were confidential and the responses were kept as anonymous. The survey was open for 6 weeks and it was voluntary to each visitor who wanted to enter the website and read the announcement.

## Statisatical analysis

The collected data and responses were coded, and statistical analysis was performed using SPSS Statistics software for Windows, Version 23.0 (IBM SPSS Statistics Windows, Version 23.0 Armonk, NY: IBM). All data were tested for normality using the Shapiro–Wilk test. Since all data were not following a normal distribution, Mann–Whitney-U test was used to explore the effect of GPA and gender on the different studied aspects. A *p* value of less than 0.05 was considered statistically significant.

Descriptive statistics were generated. Pearson’s rho test was used to examine correlations between groups. To label the strength of the association, for absolute values of rho, 0–0.19 was regarded as very weak, 0.2–0.39 as weak, 0.40–0.59 as moderate, 0.6–0.79 as strong and 0.8–1 as very strong correlation. χ^2^ Tests were used to examine the differences between categorical variables and both gender and AA-GPA.

## Results

The sample was composed of (736) students, (598) females (81.25%) and (138) males (18.75%). Statistically significant positive correlation (0.3) (weak in strength) was found between the HS-GPA and the AA-GPA (p ≤ 0.05). The significant correlations and their strengths remained the same when gender of students was taken into account.

### Career satisfaction

Electronic survey link was sent via emails to 525 dentists. Responses were collected from 408 dentists, The number of participants who viewed the survey were 408, the view rate was 77.7%. The number of participants who completed the survey and matched correctly were 333. The completion rate was 82%. 44.4% of the respondents were employed in the private sector, 15.9% unemployed, 15.4% are post-graduate students, 13.2% are employed in the public sector, and 8.5% own their private clinics. The dichotomy data which were extracted from the web-survey’s charts are described in Table 3. Other data related to scale (1–10) questions were summarized in Tables 4 and 5.

**Table 3:** The responses gathered from the web-survey’s charts for the dichotomous questions 1, 8, and 10

Dichotomy questions	Responses (n)		Responses (%)	
	Female (n = 265)	Male (n = 68)	Female 78.8%	Male 21.2%
Full time practitioner	161	53	62.7%	
Part time practitioner	104	15	37.3%	
Dentistry was first choice	163	33	59.8%	
Dentistry was not first choice	102	35	40.2%

**Table 4:** The responses gathered from the web-survey’s charts for the scale questions 3–7

	On a scale of 1–10 have you been properly trained for your work?	On a scale of 1–10 are you able to enjoy your personal life?	On a scale of 1–10 are you satisfied with your job income?	On a scale of 1–10 how much stress do you feel at work?	On a scale of 1–10 are you satisfied about your current situation?
Median	8	7	5	7	6
Mode	8	7	1	8	7
*Percentiles*					
25	7	5	3	5	4
50	8	7	5	7	6
75	9	8	7	9	8

n = 333

**Table 5:** The responses gathered from the web-survey’s charts for the scale questions 3–7

	Have you been properly trained for your work? %	Are you satisfied with your job income? %	Are you able to enjoy your personal life? %	How much stress do you feel at work? %	Are you satisfied about your current situation? %
1	1.2	17.1	4.5	1.8	10.2
2	0.3	6.3	0.6	1.2	4.8
3	1.2	8.1	4.8	3.9	6.0
4	2.7	7.5	7.5	4.2	5.1
5	7.8	16.8	12.3	15.3	16.8
6	7.2	12.9	10.8	11.4	9.9
7	20.4	16.8	21.9	12.6	20.7
8	32.7	7.8	20.7	22.5	15.0
9	16.2	4.5	10.5	12.0	6.3
10	10.2	2.1	6.3	15.0	5.1
Total	100.0	100.0	100.0	100.0	100.0

Pearson correlations and χ^2^ tests were used to measure correlations between AA-GPA as well as gender and the different tested factors (Table 6). Correlation results indicated that there was a statistically significant positive correlation between the present employment status and their AA-GPA (*p* < 0.05). There was a negative correlation between was dentistry your first choice as a profession, are you able to enjoy your life and are you satisfied with your income and the university AA-GPA. As for the correlation with gender, there was no statistically significant correlation between gender and the different tested factors as seen in Table 6. Moreover, χ^2^ test results indicated there is no relation between AA-GPA and gender and any of the tested variables (*p* > 0.05).

**Table 6:** Results of pearson correlations and χ^2^ tests along with *p* value measured at a significant level of *p* < 0.05 for gender and GPA versus all other factors

	GPA				Gender			
	Pearson correlation	*p* value	χ^2^	p	Pearson correlation	*p* value	χ^2^	p
Are you satisfied about your current situation	0.05	0.35	10.3	0.1	0.08	0.16	2.2	0.3
Are you a full time or a part time	0.02	0.73	0.4	0.9	0.15	0.01	6.9	0.008
Was dentistry your first choice as a profession	-0.05	0.3	11.6	0.01	0.11	0.05	3.7	0.05
If you could choose a different career what would it be	0.04	0.5	15.2	0.4	0.01	0.85	17.5	0.004
Where do you see yourself in 5 years	0.03	0.61	9.6	0.8	0.001	0.98	4.4	0.5
Have you been properly trained	0.02	0.67	5.9	0.4	0.04	0.50	1.1	0.6
Are you able to enjoy your life	-0.01	0.83	5.5	0.4	0.05	0.42	1.3	0.5
Are you satisfied with your income	-0.06	0.27	3.9	0.7	-0.01	0.86	0.4	0.8
How much stress you feel at work	0.08	0.16	4.5	0.6	0.07	0.23	3.1	0.2

Results of Kruskal Wallis test indicated that there was a statistically significant difference between the employment status and whether dentistry was their first choice and their GPA (*p* < 0.05).

## Discussion

The current study investigated whether there is a correlation between dental students HS-GPA and their graduating cumulative AA-GPA. The first null hypothesis was rejected and a significant positive correlation of (0.3) was found between dental students' HS-GPA and their graduating cumulative AA-GPA. Furthermore, the second null hypothesis was also rejected, since dental students' AA-GPA is significantly correlated with their CC-JS.

The body of research that has addressed the impact of preadmission factors on student success, or the achievement of professional licensure and graduation at the graduate level, is rich when analyzed across disciplines [19]. Pre-admission factors, defined broadly, are the scores or grades used to evaluate the academic and personal readiness of a prospective student to enter graduate-level specialty training [20, 21]. Achieving at a high level in preadmission factor scores is considered an excellent predictor of the likelihood for student success in their educational program [19].

When preadmission factors and selection tools are investigated separately, research supports a statistically significant correlation between HS-GPA and academic achievement and performance for students in dental and medical schools [4, 6, 7, 22, 23]. As HS-GPA is an important predictor of student AA-GPAs at health colleges [4, 22–24]. The results of our study in the School of Dentistry at University of Jordan support these findings and confirm them. However, interpreting these results has to be done carefully, since the statistically significant correlation between HS-GPA and the AA-GPA is weak (0.3). It is necessary to remember that correlations below 0.40 have limited predictive value, even if statistically significant [25]. This result is in accordance with other studies that showed that HS-GPA has limited value as a predictor of students’ performance [26], and thus necessitates using multiple criteria to better characterize the academic and psychomotor skills required for success in dental schools [24, 26].

Furthermore, there are other studies which stated clearly that the HS-GPA is an invalid predictable parameter of dental or medical students’ academic performance and success [20, 27]. In our study, the HS-GPA did not explain more than 9% of the AA-GPA variance. Thus, more variance in performance is left unexplained than explained by these selection tools [6, 25].

Other variables associated with undergraduate programs generally to college years and specifically to dental schools need to be considered when analyzing factors affecting the dental student AA-GPA [25], such as the noncognitive elements, psychomotor skills, personal characteristics, critical thinking, communication skills, teaching environment, life events and other variables taking place during college years in dental school which would affect the graduating AA-GPA rather than the HS-GPA.

While cumulative HS-GPA and graduating AA-GPA are variables which measure academic performance that depends on cognitive abilities, job performance depends on essential diverse of elements that are wider in prospective than just mathematical grades. It includes various dimensions which demand more complex set of tasks and skills composed of both cognitive and non-cognitive abilities. In health sector, in particular, it is of high significance, since it could affect the quality of patients' health care and service.

Roth et al. [28] demonstrated the presence of a relationship between academic grades and job performance through meta-analysis [28]. However, Nelson [14] argued that there were situations in which skills learned in college were not required for the practical life and skills such as social skills affect job although are not learned in college courses [14].

The link between job performance and job satisfaction was extensively studied in the literature, and a consistent relation was found between individuals’ job performance and job satisfaction regardless of the cause-effect assumptions [29–31]. On the other hand, does the motivated and high-achieving dental graduate excel at his or her career? Does he or she perform better than the norms? Is he or she satisfied with their career achievement and job situation?

The results of the current study demonstrated a significant positive correlation between AA-GPA and employment status. This result is in accordance with other studies that showed that the academic GPA is heavily weighted by many employers when going through resumes [12, 32].

On the other hand, our study demonstrated a significant negative correlation between AA-GPA and job satisfaction. In short, job satisfaction is the employee’s psychological perception of his or her job, such psychological perception comes mostly from the job itself “intrinsic satisfaction” and from the environment external to the job “extrinsic satisfaction” [33]. Indeed, extensive research was conducted and related personality traits to both job performance and job satisfaction [33–35]. Those studies found a significant positive correlation between the “Five Big” personality traits and job satisfaction [33, 36, 37].

We can argue that fresh graduate dentists with high AA-GPA may be shocked with the imperfect economical and professional reality which does not match their expectations and ambitions. As a result, the insecure situation might have yielded an emotionally unstable condition which reflected their job dissatisfaction [38]. This fact is widely presented especially in developed countries, where highly ambitious achievers are faced with multiple challenges such as unemployment, low income, and lack of job security. These dental career challenges which are noticeable in Jordan as a developing country may explain the negative correlation between dentists’ AA-GPA, their ability to enjoy life, and their dissatisfaction with their income. In addition, 34.6% and 29.5% of the respondents were looking forward to starting their post-graduate studies and open their own clinics in five years, respectively. The lack of post-graduate educational opportunities and shortage of starting capital to open and run dental business consequently might have resulted in job dissatisfaction and frustration. Those results are not in accordance with Vermeulen and Schmidt study (2008), who showed that AA-GPA had a positive relationship with earnings and job satisfaction in the initial phase of the graduates’ working career [39]. Additionally, these results also contradict Thomas results (2000) who noted that alumni with high grades during their studies were later more successful in terms of earnings [40]. The economic status as one facet of multi facet career challenges for dentists in Jordan which may explain these contradicting results. The differences in the setting of the study; the place of the study (country), the timing (year), and the measurement scale might have contributed to the differences in the results.

The results of the present study rejected the first null hypothesis; were there is a significant positive correlation between high school cumulative HS-GPA and graduating AA-GPA of dental students. The second null hypothesis was rejected too; there is a significant positive correlation between dental students' AA-GPA and their career choices CC-JS. These results mandate additional prospective studies with multivariate analysis will yield more informative results than retrospective studies providing deeper insight into dominant factors which affect academic performance. More studies are required to elucidate trends and predictors for successful academic performance, as well as to plan and evaluate organizational development and curriculum structure in dental education. Moreover, these results are significant because they identify the extent to which the preadmission criterion applied can predict successful dental graduates and the extent to which the academic performance can predict successful dentists. This important information is provided for policy makers looking to update their dental school admissions procedures in response to the changing dental educational landscape.

Since this is a retrospective study, a major limitation is the incapability of studying other factors that could affect the graduating AA-GPA of dental students. It is well known to the researchers through their academic experience that there are variables other than HS-GPA contribute to the graduating AA-GPA. Those variables include life and social events, peer pressure, environmental support, social and financial elements, and personal characteristics and traits.

## Conclusions

The present study demonstrated the followings:

1. GPA remains an important performance predictor across the dental curriculum. However, it is important to remember that the dental school admission process exists not only to select the candidates who are most likely to do well in dental school, but also to select a diverse body of capable students, giving rise to competent practitioners who can best serve dental profession and patients.
2. Dental students' AA-GPA affects their career choices as well as their job satisfaction CC-JS.

## Supplementary Information

**Additional file 1**. Survey consists of 12 questions covering demographic and professional background characteristics and attitude towards job to assess career satisfaction.

## Data Availability

The datasets used and/or analyzed during the current study are available from the corresponding author on reasonable request. The datasets analyzed during the current study are not publicly available due to limitations of ethical approval involving the student data and anonymity but are available from the corresponding author on reasonable request.

## Abbreviations

GPA: Grade Point Average
HS-GPA: High School Grade Point Average
DAT: Dental Admission Test
GAMSAT: Graduate Australian Medical School Admissions Test
SAT1: Saudi National Aptitude Exam
SAT2: Saudi National Achievement Exam
AA-GPA: Academic Achievement
CC-JS: Career Choice and Job Satisfaction
FDREC: Faculty of Dentistry Research and Ethics Committee
IRB: Institutional Review Board at the University of Jordan
