# Supplementary material for: The predictive value of high school grade point average to academic achievement and career satisfaction of dental graduates
Source: BMC Oral Health. 2021 Jun 12;21:300. doi: 10.1186/s12903-021-01662-5 (PMC8196503; doi:10.1186/s12903-021-01662-5)
Supplement: Supplementary file 1 — Additional file 1. Survey consists of 12 questions covering demographic and professional background characteristics and attitude towards job to assess career satisfaction. [file 12903_2021_1662_MOESM1_ESM.docx]

**The Predictive Value of High School Grade Point Average to Academic Achievement and Career Satisfaction of Dental Graduates**

**Appendix (1)**

Q1: Gender:

1. Male
2. Female

Q2: Currently you are:

1. Unemployed
2. Employed in the private sector
3. Employed in the public sector
4. Owner of a clinic
5. Post-graduate student
6. Teacher assistant at a university
7. Non clinical dental related field of work
8. Non dental related field of work

Q3: On a scale of 1-10 are you satisfied about your current situation?

(1 is no and 10 is strongly yes)

- 1 – 2 – 3 – 4 – 5 – 6 – 7 – 8 – 9 – 10

Q4: On a scale of 1-10 have you been properly trained for your work?

(1 is no and 10 is strongly yes)

- 1 – 2 – 3 – 4 – 5 – 6 – 7 – 8 – 9 – 10

Q5: On a scale of 1-10 are you able to enjoy your personal life?

(1 is no and 10 is strongly yes)

- 1 – 2 – 3 – 4 – 5 – 6 – 7 – 8 – 9 – 10

Q6: On a scale of 1-10 are you satisfied with your job income?

(1 is no and 10 is strongly yes)

- 1 – 2 – 3 – 4 – 5 – 6 – 7 – 8 – 9 – 10

Q7: On a scale of 1-10 are you under great pressure at work?

(1 is no and 10 is strongly yes)

- 1 – 2 – 3 – 4 – 5 – 6 – 7 – 8 – 9 – 10

Q8: Are you a full-time or part-time practitioner?

1. Full time practitioner (> 48hours a week).
2. Part time practitioner (< 30hours a week).

Q9: If you are a part-time practitioner or you are not practicing clinical dentistry, the main reason is because:

1. You are not interested in practicing dentistry
2. You are caring for children and family
3. Your health is compromised
4. You prefer to spend your time on other stuff
5. You couldn’t find a job and cannot open your own practice
6. Other reasons

Q10: Was dental profession your first choice as a professional career when you applied to the university?

1. Yes
2. No

Q11: If it was not, if you could go back in time and change your profession what would you choose?

1. Medicine
2. Other health related career: pharmacy, nursing, physiotherapy... etc.
3. Art stream: languages, law, politics… etc.
4. Others

Q12: Where do you see yourself in 5 years?

1. Working at the same job
2. Opening my own practice
3. Quitting dentistry and work in another field
4. Starting my post-graduate studies
5. Teaching at the university
